# Anxiety levels and physiological responses during top-rope and lead climbing: a cross-sectional study among sport climbers

**DOI:** 10.1186/s13102-025-01493-9

**Published:** 2025-12-30

**Authors:** Natalia Swinarska, Patrycja Krężel, Aleksandra Hapka, Anna Jurczak, Sylwia Wieder-Huszla

**Affiliations:** 1 Department of Observational Infectious Diseases, Tropical Diseases and Acquired Immunodeficiencies, Independent Public Voivodship Hospital, ul. Arkońska 4, Szczecin, 71-455 Poland; 2 Department of Specialized Nursing, Pomeranian Medical University in Szczecin, Szczecin, 71-210 Poland; 3 Students’ Scientific Society of Department of Specialized Nursing, Pomeranian Medical University in Szczecin, Szczecin, 71-210 Poland

**Keywords:** Anxiety, Stress, Sport climbing, Extreme sports

## Abstract

**Objectives:**

Sport climbing presents an exceptionally interesting research field for issues related to anxiety and stress. This discipline, classified as an extreme sport, requires participants not only to be in very good physical condition, but also to be mentally resilient and able to cope with high emotional pressure. Theoretical models of self-efficacy and psychophysiological load suggest that individual characteristics (e.g., gender, BMI) and situational factors (e.g., belay type) may shape climbers’ emotional responses by influencing perceived control, physical strain, and risk appraisal. The aim of the study was to assess the level of anxiety experienced by sport climbers.

**Design:**

This was a cross-sectional study.

**Method:**

The study was performed among 100 people practising sport climbing on climbing walls in three large Polish cities. The study was conducted between November 2023 and January 2024. The research was divided into three parts: a structured interview, measurement of anthropometric and vital signs parameters before and after climbing. A self-designed survey questionnaire and a standardised tool, i.e. STAI X-1 and STAI X-2, were used to collect empirical data.

**Results:**

The study showed that climbers experienced significantly more anxiety during lead climbing (40.1 ± 9.2) than when top-rope climbing (33.1 ± 6.3). Anxiety as a state during climbing with a bottom belay was observed at a high level in 46% of the participants.

**Conclusions:**

Most participants experienced anxiety during sport climbing. Their anxiety was reflected by physiological changes. The determining factors for the participants’ feelings of anxiety during climbing were gender, BMI and climbing method. In the study group, climbers experienced significantly higher levels of anxiety when climbing with a lead climbing than a top-rope climbing.

## Introduction

Anxiety and stress are integral parts of human existence, especially in situations that cause emotional tension. Stress theory, developed by researchers such as Cannon W., Selye H. or Lazarus R. and Folkman S., provides important tools for analysing the physiological and psychological responses of the body to these states [1].

Anxiety is an emotional state that manifests itself as uneasiness, feelings of tension or threat. It is defined in the literature as ‘a negative emotional state that is a person’s reaction to an implicit (imagined) threat, expressing itself through feelings of tension, anxiety’ [1–3]. It is often equated with fear, the source of which is real, immediate danger. It is recognised that during the experience of anxiety there is ‘somatic hyperactivity’, i.e. sweating, shallow and accelerated breathing, palpitations or high heart rate [1–3]. Such symptoms are the result of functioning of two different systems in the human body. The first is responsible for the secretion of cortisol, which under normal conditions is regulated by a negative feedback mechanism that prevents excessive production of this hormone. When stress is experienced, this mechanism is weakened, resulting in increased cortisol in the body. The second key mechanism when experiencing stress is the stimulation of the body to secrete adrenaline and noradrenaline, which has a significant effect on the cardiovascular system. Heart rate increases and there is a noticeable rise in blood pressure. The cooperation of these two systems prepares human body for increased alertness, endurance, and high effort. The aim of it is to prepare the individual to function under extraordinary conditions [1, 2, 4].

Sport climbing is an exceptionally interesting research topic regarding issues related to anxiety and stress. This discipline, classified as an extreme sport, requires participants not only to be in very good physical condition, but also to be mentally resilient and able to cope with high emotional tension. Climbers often exhibit spontaneous stress responses, including vocalization or abrupt movements [5–7]. During climbing, the type of belay is an important element. The simplest categorisation of rope belaying is top rope climbing (TR) and bottom rope climbing. Top-rope climbing is characterised by the rope passing through a belaying site that is above the climber. This results in the climber remaining almost in the same place where they fell off. This technique represents the most basic form of artificial-wall belaying on climbing walls [8, 9]. Climbing with a bottom belay requires more experience from both the climber and the belayer. It involves the person intending to climb attaching themselves to the rope and, on ascending the route, clipping the rope into the belay points. If the belay points are fixed in the rock or on the climbing wall (rings, bolts, etc.). This configuration corresponds to standard sport-climbing practice. It is practised on climbing walls and rocks. If belay points are placed in the rock by the climber while climbing, then this type is called trad climbing (climbing on one’s own belay) [8].

Climbing as a sport is becoming increasingly popular every year. There are more and more artificial climbing walls being built, attracting a lot of interest. The introduction of sport climbing to the Olympic Games programme in 2020 in Tokyo has highlighted its global significance. The aim of this study was to analyse feelings of anxiety during sport climbing.

## Methods

### Study design

The study group consisted of 100 individuals (35 women and 65 men) practising climbing in three Polish cities: Szczecin, Kraków and Łódź. Participants were recruited on a voluntary basis at local climbing gyms. Informed consent was obtained from all participants prior to inclusion in the study. Research was conducted in accordance with the Declaration of Helsinki and approved by Bioethics Committee of Pomeranian Medical University in Szczecin (Resolution No. KB-0012/46/01/2013). Inclusion criteria for the study were: age of 18 years or older, consent to participate in the study, good psychophysical condition, ability to climb with two types of belays, i.e. top-rope and bottom belay.

A typical testing session followed a standardized timeline. Upon arrival, each participant was informed about the study procedures and provided written informed consent. Next, participants completed the demographic and climbing questionnaire as well as the STAI-X2 (trait anxiety). After a 5-minute seated rest, baseline measurements were taken, including height, weight and BMI, resting blood pressure, and resting heart rate. A POLAR H10 heart-rate sensor was then fitted for continuous monitoring.

Each participant completed two climbing routes in a fixed order: first with top-rope belay, and then with lead belay. The difficulty of both routes was matched to the participant’s maximal climbing level, ensuring comparable physical, technical, and emotional demands. During each climb, heart rate was recorded continuously. Immediately after the first climb, blood pressure and heart rate were measured, and participants completed the STAI-X1 assessing state anxiety related to that climb. A short rest period of approximately 10 min followed.

Participants then completed the second route (lead belay), again with continuous heart rate monitoring. Immediately after this climb, blood pressure and heart rate were measured once more, and participants completed the second STAI-X1 referring to the lead-belay attempt. At the end of the session, the heart-rate sensor was removed, any adverse events recorded, participant debriefed and thanked. Data are checked for completeness.

### Research tools

The study protocol comprised three components: (1) a structured interview; (2) anthropometric and physiological measurements; and (3) completion of two climbing routes with different belay types. A self-designed survey questionnaire (regarding basic sociodemographic data, climbing preferences, and awareness of experiencing anxiety while climbing) and a standardised research tool - STAI, were used to collect empirical data.

#### State Trait Anxiety Inventory (STAI)

The State–Trait Anxiety Inventory (STAI) was used to assess anxiety. It consists of two subscales: the first (STAI X-1) is used to assess anxiety as a state (transient, situationally conditioned) and the second (STAI X-2) allows assessment of anxiety as a trait (understood as a relatively permanent personality trait). Each subscale consists of 20 items. The score for each subscale can range from 20 to 80 points; higher scores indicate higher levels of anxiety. In this study, anxiety levels were interpreted based on raw scores, without conversion into sten values, to ensure clarity for international readers. In addition to the STAI, a self-designed questionnaire was used to collect contextual information related to climbing. This tool has not been formally validated, which represents a limitation of the present study [10].

#### Anthropometric measurements

Anthropometric measurements were taken on an after an overnight fast, in light clothing, without shoes, after bladder emptying, using an electronic scale with an altimeter. Based on the obtained data, body mass index (BMI) was calculated - values of 18.5–24.9 kg/m2 were considered normal, overweight was defined with BMI values ranging from 25.0 to 29.9 kg/m2 and obesity was identified with a BMI of 30 kg/m2 or more.

#### Measurement of vital parameters

Heart rate and blood pressure were measured before and after climbing. Additionally, the climber had their heart rate measured continuously during the climb with a simultaneous determination of the highest value of this parameter. Blood pressure was measured using a medical blood pressure monitor from INCOMEDICA model CM EU and a Littmann Classic IIITM stethoscope. Heart rate was measured continuously using a POLAR H10 sensor placed on the climber’s chest.

### Statistical analysis

Non-parametric tests were used for statistical analyses: the Mann-Whitney u-test - to verify the significance of the differences in two groups, the Kruskal-Wallis test - to evaluate the significance of the differences in at least three groups, and the Spearman rank correlation coefficient significance test - to analyse the correlation between measurable variables. Assumed significance level was *p* < 0.05.

## Results

### Characteristics of the study group

The study included 100 climbers aged 19–65 years. Most participants were male, professionally active, and of normal body weight. Anthropometric and demographic characteristics are presented in Table 1.

**Table 1:** Participant demographics, anthropometric measurements, and climbing-related variables

Variable	Women (*n* = 35)	Men (*n* = 65)	Total (*n* = 100)
Sex (%)	35.0%	65.0%	100%
Age (years) mean ± SD (median; min–max)	29.1 ± 6.4 (27; 19–44)	29.0 ± 7.8 (28; 20–65)	29.0 ± 7.3 (27.5; 19–65)
Marital status (%)	single 48.6% married 40.0% cohabiting 11.4% divorced 0%	single 66.2% married 21.5% cohabiting 10.8% divorced 1.5%	single 60% married 28% cohabiting 11% divorced 1%
Place of residence (%)	village 5.7% town 10–100k: 8.6% city > 100k: 85.7%	village 4.6% town 10–100k: 18.5% city > 100k: 76.9%	village 5% town 10–100k: 15% city > 100k: 80%
Education (%)	secondary 31.4% higher 68.6%	secondary 46.2% higher 53.8%	secondary 41% higher 59%
Occupational activity (%)	employed 80% student 34.3% other 2.9%	employed 75.4% student 35.4% pension 1.5% other 1.5%	employed 77% student 35% other 2%
Height (cm) mean ± SD (median; min–max)	168.4 ± 5.0 (169; 157–178)	180.5 ± 6.0 (180; 170–195)	176.3 ± 8.1 (176.5; 157–195)
Weight (kg) mean ± SD (median; min–max)	60.8 ± 5.4 (61; 51–74)	77.8 ± 8.5 (78; 59–105)	71.9 ± 11.1 (71; 51–105)
BMI mean ± SD (median; min–max)	21.45 ± 1.62 (21.61; 18.34–25.01)	23.87 ± 2.32 (23.30; 20.15–31.14)	23.02 ± 2.39 (22.81; 18.34–31.14)
BMI categories (%)	underweight 2.9% normal 94.3% overweight 2.9% obesity I 0%	underweight 0% normal 76.9% overweight 18.5% obesity I 4.6%	underweight 1% normal 83% overweight 13% obesity I 3%

*SD* Standard deviation, *BMI* Body Mass Index, *k* Thousand inhabitants

### Level of anxiety experienced while climbing

In the surveyed group, anxiety as a state during climbing with a belay was observed at a high level in 46% of the participants. Average and low levels of anxiety during climbing with a bottom belay were obtained by 33% and 21% of the climbers, respectively. On the other hand, climbing with a top-rope belay, was associated with an average (47%) and low (44%) level of anxiety. Only 9% of the respondents experienced this emotion in high intensity. There was a statistically significant difference in anxiety between those climbing with a top vs. bottom belay (*p* < 0.0001). Anxiety as a state was significantly higher when climbing with a bottom belay (Table 2).

**Table 2:** Analysis of anxiety severity according to the type of belay used during climbing

Type of belay	n	STAI X-1 State - Anxiety					Z	p
		M	SD	Mdn	Min	Max		
Top-rope belay	100	33.1	6.3	33.0	20	65	8.57	< 0.0001
Bottom belay	100	40.1	9.2	39.0	21	70		

M – mean, SD – standard deviation, Mdn – median, Z – z score, *p* < 0.05

The next part of the study was to assess the influence of selected variables on the participants’ perceived level of anxiety. The analysis did not show correlation between age, marital status, education level or climbing experience and anxiety levels. However, such a correlation was found in relation to gender. The results showed that women were characterised by significantly higher levels of anxiety both as a Trait-Anxiety (*p* = 0.0001) and as State-Anxiety during climbing with a top (*p* = 0.0006) and bottom belay (*p* = 0.0001) - Table 3.

**Table 3:** Analysis of severity of anxiety in relation to gender of the participants

Gender	*n*	STAI X-1					Z	*P*
		Climbing with a top-rope belay State-Anxiety						
		M	SD	Mdn	Min	Max		
Female	35	35.9	6.7	35.0	23	65	3.45	0.0006
Male	65	31.6	5.6	32.0	20	46		

	**n**	**Climbing with a bottom belay ** **State-Anxiety**						
		**M**	**SD**	**Mdn**	**Min**	**Max**		
Female	35	44.7	9.1	43.0	30	70	3.80	0.0001
Male	65	37.6	8.3	37.0	21	63		

	**n**	**STAI X-2 ** **Trait-Anxiety**						
		**M**	**SD**	**Mdn**	**Min**	**Max**		
Female	35	44.8	9.6	42.0	29	67	3.80	0.0001
Male	65	37.8	8.5	36.0	23	68		

M – mean, SD – standard deviation, Mdn – median, Z – z score, *p* < 0.05

Another variable that proved to be significant was BMI. Findings indicated that, participants with normal weight or underweight were characterised by significantly higher levels of anxiety both as Trait-Anxiety (*p* = 0.0043) and State-Anxiety during climbing with top (*p* = 0.0249) and bottom belay (*p* = 0.0350) - Table 4.

**Table 4:** Analysis of severity of anxiety in relation to BMI of the participants

**BMI**	**n**	**Climbing with a top belay ** **State-Anxiety (points)**					**Z**	**p**
		**M**	**SD**	**Mdn**	**Min**	**Max**		
Normal/underweight	84	33,7	6,4	34,0	22	65	2,24	0,0249
Overweight/obesity	16	29,9	5,2	31,0	20	38		

	**n**	**Climbing with a bottom belay ** **State-Anxiety (points)**						
		**M**	**SD**	**Mdn**	**Min**	**Max**		
Normal/underweight	84	41,0	9,4	40,5	25	70	2,11	0,0350
Overweight/obesity	16	35,8	6,2	36,5	21	50		

	**n**	**Trait-Anxiety (points)**						
		**M**	**SD**	**Mdn**	**Min**	**Max**		
Normal/underweight	84	41,4	9,6	39,5	25	68	2,86	0,0043
Overweight/obesity	16	34,3	6,2	34,5	23	48		

M - mean, SD – standard deviation, Mdn – median, Z – z score, p<0,05

In the next stage of the study, an attempt was made to evaluate STAI X-1 and STAI X-2 scores with the participants’ subjectively perceived anxiety. Findings indicated that those who characterised themselves as subjectively anxious had significantly higher levels of anxiety both as a Trait-Anxiety (*p* = 0.0340) and State-Anxiety when climbing with top (*p* = 0.001) and bottom belay (*p* = 0.0001) - Table 5.

**Table 5:** Analysis of severity of anxiety in relation to subjective feeling of anxiety during climbing in participants

Incidence of climbing anxiety	n	Climbing with a top-rope belay State-Anxiety (points)					Z	P
		M	SD	Mdn	Min	Max		
Yes	68	34.5	6.1	34.0	22	65	3.25	0.0012
No	32	30.2	5.8	28.5	20	43		

	**n**	**Climbing with a bottom belay** **State-Anxiety (points)**						
		**M**	**SD**	**Mdn**	**Min**	**Max**		
Yes	68	42.3	8.8	41.0	25	70	3.83	0.0001
No	32	35.5	8.2	34.5	21	57		

	**n**	**Trait-Anxiety (points)**						
		**M**	**SD**	**Mdn**	**Min**	**Max**		
Yes	68	41.8	10.0	38.0	26	68	2.12	0.0340
No	32	37.0	7.2	36.5	23	55		

M - mean, SD – standard deviation, Mdn – median, Z – z score, *p* < 0.05

Analysis of systolic and diastolic blood pressure results showed statistically significant differences. Post-climb systolic pressure was significantly higher in the participants during both climbing with an upper (*p* < 0.0001) and lower belay (*p* = 0.0003). Similarly, diastolic pressure after climbing was significantly higher during climbing with top (*p* < 0.0001) and bottom belay (*p* = 0.00022). The evaluation of heart rate measurements after climbing confirmed significant statistical differences during both climbing with a top (*p* < 0.0001) and bottom belay (*p* < 0.0001). The analysis showed that the correlation of maximum heart rate between climbing with top and bottom belay was also statistically significant (*p* < 0.0001). When climbing with a bottom belay, the maximum heart rate was significantly higher - Table 6.

**Table 6:** Analysis of blood pressure and heart rate measurements during climbing

Measurement time	n	Climbing with a top-rope belay Systolic blood pressure (mmHg)					Z	p
		M	SD	Mdn	Min	Max		
5 min before climbing	100	128.2	11.1	128.0	108	153	7.06	< 0.0001
5 min after climbing	100	134.7	11.2	133.0	112	180		

	**n**	**Climbing with a bottom belay ** **Systolic blood pressure (mmHg)**						
		**M**	**SD**	**Mdn**	**Min**	**Max**		
5 min before climbing	100	135.1	10.6	135.0	110	157	3.59	0.0003
5 min after climbing	100	137.5	10.7	137.5	115	175		

	**n**	**Climbing with a top-rope belay ** **Diastolic blood pressure (mmHg)**						
		**M**	**SD**	**Mdn**	**Min**	**Max**		
5 min before climbing	100	80.4	8.0	80.0	66	100	5.60	< 0.0001
5 min after climbing	100	84.4	7.1	85.0	70	110		

	**n**	**Climbing with a bottom belay ** **Diastolic blood pressure (mmHg)**						
		**M**	**SD**	**Mdn**	**Min**	**Max**		
5 min before climbing	100	83,8	7,3	83,5	69	97	3,06	0,0022
5 min after climbing	100	85,9	7,5	87,0	70	100		

	**n**	**Climbing with a top-rope belay ** **Heart rate**						
		**M**	**SD**	**Mdn**	**Min**	**Max**		
5 min before climbing	100	89.8	14.2	92.0	62	120	5.43	< 0.0001
5 min after climbing	100	94.6	12.5	97.5	68	120		

	**n**	**Climbing with a bottom belay ** **Heart rate**						
		**M**	**SD**	**Mdn**	**Min**	**Max**		
5 min before climbing	100	95.0	11.4	96.5	72	121	5.35	< 0.0001
5 min after climbing	100	98.5	11.2	100.0	74	126		

**Type of belay**	**n**	**Maximum heart rate**						
		**M**	**SD**	**Me**	**Min**	**Max**		
Top-rope belay	100	167.3	9.8	168.0	122	189	8.47	< 0.0001
Bottom belay	100	179.1	7.9	179.0	156	197		

M - mean, SD – standard deviation, Mdn – median, Z – z score, *p* < 0.05

**Fig. 1:**
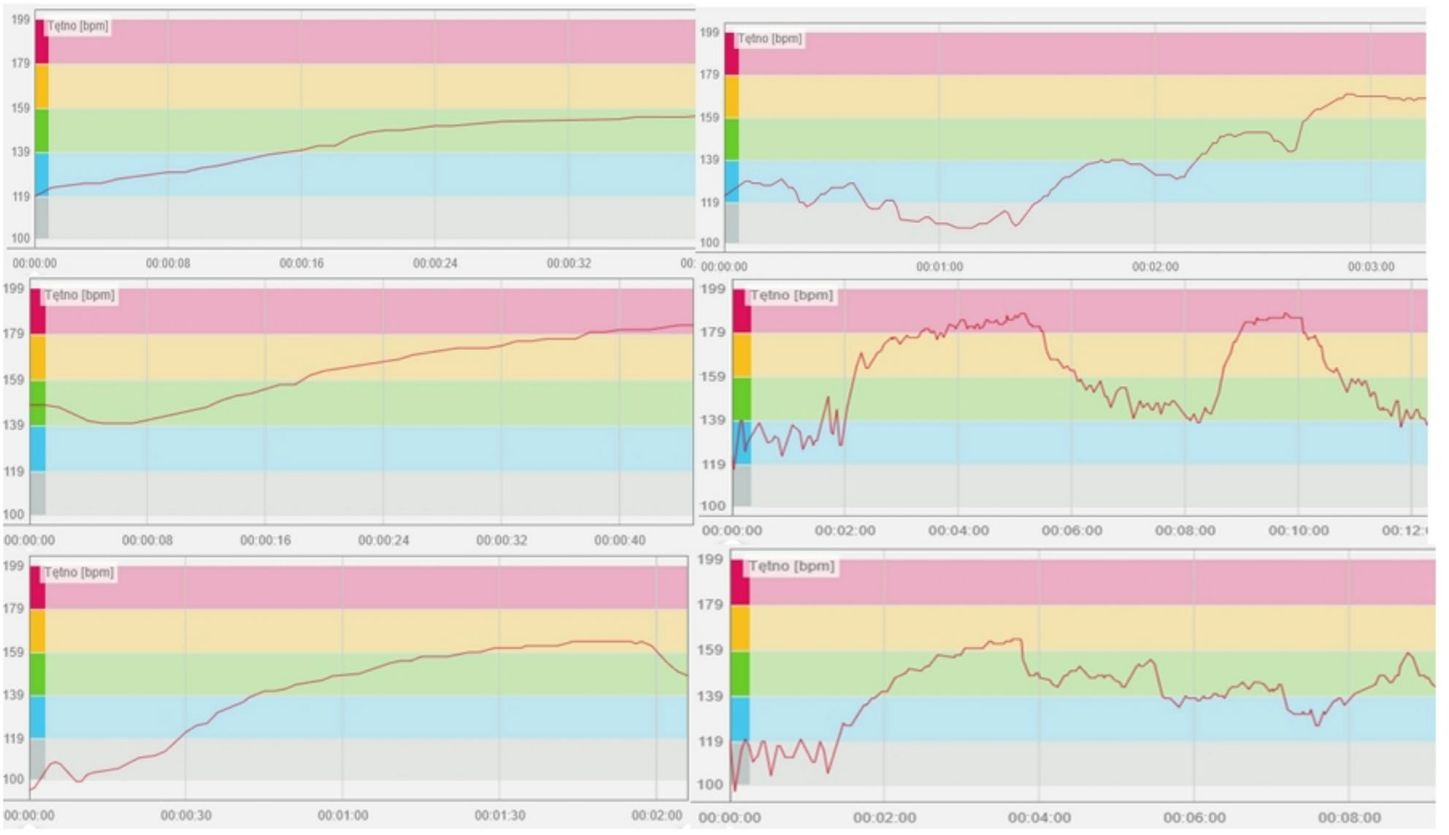
Heart rate waveform during top-rope climbing (left) and with bottom belay (right)

An analysis of the heart rate graphs during climbing revealed distinct patterns for different belay types. When climbing with a top-rope belay. the climber’s heart rate increased steadily and linearly with effort, reflecting a consistent physiological response to the physical demands of the route. In contrast, climbing with a bottom belay produced a more irregular heart rate waveform, characterized by sharp spikes. These fluctuations appeared to correspond to moments when the climber was clipping the rope or navigating riskier movements, which could increase the likelihood of falls Fig. 1.

## Discussion

As a multidisciplinary sport, climbing requires focusing on the psychological aspect, as it is crucial to understand the whole process that takes place in the body during this activity. Anxiety as a state can be defined as a transient, situational feeling of distress that changes in intensity depending on the circumstances. While experiencing anxiety, there is an increased activity of the sympathetic nervous system, which usually causes disturbances in the body’s homeostasis. During climbing, a significant correlation has been observed between perceived anxiety and the climber’s choice of belay [11, 12]. Czermak, et al. conducted a study which proved that although the belay systems used in recreational climbing provide participants with complete safety, the method of belaying not only influences the level of experienced anxiety, but also climber’s effectiveness at completing the route. Dynamic belaying induced a higher level of anxiety in the climbers (the participants scored on average 49.36 points on the STAI scale in dynamic climbing and 46.9 points in static climbing) and their heart rate was higher than in static belaying. In addition, being secured by dynamic belaying 26% of the participants did not complete the climbing route, where with static belaying 100% of the climbers completed it [6]. In this study, similar results were obtained - the mean level of State-Anxiety during top-rope climbing was 33.1 ± 6.3 points, while in the participants climbing with a bottom belay it was 40.1 ± 9.2 points. Almost half of the climbers had a high level of anxiety when climbing with a bottom belay, while the same level of anxiety when top-rope climbing occurred in only less than 10% of the participants. This represents a substantial difference in anxiety levels., which is reflected in the way the climbers behave during climbs. In the our study, differences in heart rate measurements were also noted: stressful situations during climbing, i.e. performing so-called ‘pitches’, climbing over a ‘pitch’ or traversing, triggered anxiety and thus led to an increase in heart rate measurements.

An analysis of the literature shows that gender determines the engagement in extreme activities, as men are far more likely to participate in such sports and exhibit risky behaviour. Women are observed to be more cautious when taking part in extreme sports, which may suggest that they experience considerably more anxiety [13, 14]. Bielec et al. analyzed anxiety levels in divers showing that diving also increases anxiety, especially in women. According to the authors, such emotions can cause panic behaviour [15]. Although diving is a different sport, it is classified as an extreme sport too. Our study on climbers also confirmed that women had significantly higher levels of anxiety regardless of the belay used. These findings suggest that anxiety mechanisms may share common features across various extreme sports. However, the small number of papers in this area leaves a need for further research [16, 17].

A group of researchers led by Frenkel conducted an experiment that tested the psychological and physiological stress response during falling off a climbing wall and a three-meter fall. The results showed that the psychological stress response was significantly intensified, and the level of perceived anxiety strongly increased. The authors observed that the heart rate rose as soon as the participant was explained the aim of the experiment and during the task [18]. In this study it was observed that the heart rate increased during difficult moments for the climbers, where the risk of falling off was very high. It can be concluded that it is not only climbing itself that causes anxiety, but also the possibility of falling off and so-called fall in critical situations [5, 18].

Recent international research supports these findings and provides insights into mechanisms of stress modulation. Lee, Maisarah, and Wong demonstrated that anxiety sensitivity and stress appraisal are key predictors of performance anxiety in athletes, emphasizing the role of cognitive appraisal and perceived control [19]. Zahedi et al. showed that training interventions combining psychological and skill-based approaches can improve mood and performance under pressure. These studies suggest that structured psychological preparation and exposure to challenging situations can help athletes manage anxiety and stress, which is relevant for climbing practice [20].

The literature shows that climbing is a sport that can cause highly emotional reactions. Precision, coordination and keeping the error margin to a minimum are essential. The issue of mental state in sport climbing requires further research and analysis. It is a discipline that has developed significantly in recent years, and there has been a noticeable increase of interest in it. Sport climbing is characterized by a certain uniqueness, in which the synergy of physical and mental factors is fundamental.

## Limitations

This study has several limitations that should be acknowledged. First, its cross-sectional design does not allow causal inferences regarding the relationships between belay type, physiological responses, and anxiety levels. Second, the sample was obtained through convenience sampling from selected climbing gyms, which may limit the generalizability of the findings to other populations such as outdoor climbers, elite athletes, or climbers from different regions. Third, the study relied on self-report measures, which may be subject to recall bias or subjective misinterpretation, particularly in the assessment of anxiety and climbing-related perceptions. Fourth, the analysis did not control for potential confounding variables such as training experience, climbing grade, personality traits, or fear of falling, and no correction for multiple comparisons was applied, which may increase the risk of type I error. Future research should use longitudinal or experimental designs, more diverse sampling strategies, validated psychological tools, and consider confounding factors to strengthen causal interpretation and external validity. Due to the lack of access to raw, individual-level data, effect sizes and additional correlation analyses could not be calculated, and the statistical interpretation is therefore limited to the aggregated values available.

## Conclusions

Sport climbing induces measurable anxiety in most participants, reflected in physiological responses such as increased heart rate, sweating, and trembling. This anxiety appears to be influenced by both individual characteristics (gender and BMI) and situational factors, particularly the type of belay, with bottom belay increasing perceived risk and uncertainty. These findings suggest that anxiety during climbing is shaped by the interplay of physiological load, perceived risk, and self-efficacy in managing climbing demands.

Understanding these mechanisms has practical implications. Coaches and climbers can benefit from structured psychological skills training, including fear management techniques and gradual exposure to challenging climbing situations, to enhance coping strategies and reduce anxiety. Regular psychological assessments can help identify individuals with heightened anxiety, allowing tailored interventions. Moreover, fostering open discussions about fear within the climbing community can normalize these experiences, support resilience, and prevent performance limitations or injury.

## Practical implication

- Fear and anxiety experienced during climbing, especially in lead climbing situations, may hinder performance and increase risk. Coaches should integrate psychological skills training into regular training routines to help climbers manage fear responses.
- Structured and progressive exposure to fear-inducing situations (e.g., controlled lead falls, climbing above protection) can help climbers build tolerance and reduce anxiety over time.
- Regular psychological assessments can help identify climbers with heightened anxiety levels, allowing for individualized mental training plans tailored to their needs and experiences.
- Within the climbing community, promoting open discussions around fear can shift the perception from weakness to growth, helping climbers build resilience and prevent burnout or injury caused by avoidance or overexertion.

## Data Availability

The datasets used and/or analysed during the current study are available from the corresponding author on reasonable request.

## Abbreviations

BMI: Body Mass Index
STAI X: 1-State-Trait Anxiety Inventory Scale 1
STAI X: 2-State-Trait Anxiety Inventory Scale 2
